# The *Plasmodium falciparum* male gametocyte protein P230p, a paralog of P230, is vital for ookinete formation and mosquito transmission

**DOI:** 10.1038/s41598-018-33236-x

**Published:** 2018-10-08

**Authors:** Catherin Marin-Mogollon, Marga van de Vegte-Bolmer, Geert-Jan van Gemert, Fiona J. A. van Pul, Jai Ramesar, Ahmad Syibli Othman, Hans Kroeze, Jun Miao, Liwang Cui, Kim C. Williamson, Robert W. Sauerwein, Chris J. Janse, Shahid M. Khan

**Affiliations:** 1 Leiden Malaria Research Group, Parasitology, Leiden University Medical Center (LUMC), Leiden, The Netherlands; 2 Department of Medical Microbiology, Radboud University Medical Center, Nijmegen, The Netherlands; 3 Faculty of Health Sciences, Universiti Sultan Zainal Abidin, Terengganu, Malaysia; 4 Department of Entomology, The Pennsylvania State University, University Park, Pennsylvania, United States; 5 Microbiology and Immunology Department, Uniformed Services University of the Health Sciences, Bethesda, Maryland United States

**Keywords:** Parasite biology, Molecular biology

## Abstract

Two members of 6-cysteine (6-cys) protein family, P48/45 and P230, are important for gamete fertility in rodent and human malaria parasites and are leading transmission blocking vaccine antigens. Rodent and human parasites encode a paralog of P230, called P230p. While P230 is expressed in male and female parasites, P230p is expressed only in male gametocytes and gametes. In rodent malaria parasites this protein is dispensable throughout the complete life-cycle; however, its function in *P*. *falciparum* is unknown. Using CRISPR/Cas9 methodology we disrupted the gene encoding *Pfp230p* resulting in *P*. *falciparum* mutants (*Pf*Δ*p230p*) lacking P230p expression. The *Pf*Δ*p230p* mutants produced normal numbers of male and female gametocytes, which retained expression of P48/45 and P230. Upon activation male *PfΔp230p* gametocytes undergo exflagellation and form male gametes. However, male gametes are unable to attach to red blood cells resulting in the absence of characteristic exflagellation centres *in vitro*. In the absence of P230p, zygote formation as well as oocyst and sporozoite development were strongly reduced (>98%) in mosquitoes. These observations demonstrate that P230p, like P230 and P48/45, has a vital role in *P*. *falciparum* male fertility and zygote formation and warrants further investigation as a potential transmission blocking vaccine candidate.

## Introduction

The s48/45 domain 6-cysteine (6-cys) family of *Plasmodium falciparum* proteins is a small family with 14 members that show stage-specific expression throughout the parasite life cycle and most members localize at the parasite surface^1^. Most members have critical roles in parasite development, either in the vertebrate host or in the mosquito vector, and several members are leading targets for malaria vaccines. Four members, P48/45, P47, P230 and P230p are specifically expressed in the sexual stages of the parasite and are encoded by 2 paralogous pairs of genes. Immune responses directed against the proteins P48/45 and P230 can prevent parasite transmission through the mosquito and these antigens are being actively pursued as so called transmission blocking vaccines^2–4^. Using specific antibodies and rodent and human parasite mutants lacking P48/45 and P230 it has been shown that both proteins are crucial for efficient transmission through mosquitoes^5–7^. In the rodent parasite *P*. *berghei* these proteins are expressed at the surface of male gametes and are critical for attachment of male gametes to female gametes^5^. In *P*. *falciparum* these proteins are expressed in both male and female gametocytes/gamete^8–10^. *P*. *falciparum* P230 has been shown to play a critical role in interactions of male gametes with red blood cells (RBC). In mutants lacking P230 expression the characteristic clusters of uninfected red blood cells that form around male gametes, so-called exflagellation centres, are absent^7^. This ‘loss of exflagellation centres’ phenotype was not observed for equivalent *P*. *berghei* mutants lacking P230 expression^5^. In *P*. *berghei* and *P*. *falciparum* the paralog of P48/45, the female specific P47 protein is located on the surface of female gametes, zygotes and ookinetes^11^. P47 is important in protecting ookinetes from the mosquito’s complement-like immune response in both rodent and human malaria species^12–14^. In addition, *P*. *berghei* P47 plays an essential role in the attachment and recognition of the female gamete by the male gamete^5,12^. In contrast, *P*. *falciparum* P47 does not play such a crucial role in gamete fertilization^11^. These observations indicate that differences exist in the precise function of the sex-specific 6-Cys members between human and rodent malaria species.

In two rodent *Plasmodium* species the paralog of P230, the male-specific P230p protein, appears to be dispensable throughout the parasite’s complete life cycle^5,15,16^. *P*. *berghei* and *P*. *yoelii* mutants lacking expression of P230p can develop in the vertebrate host and in the mosquito vector without a discernible phenotype and *p230p* knock-out parasites manifest a wild type parasite phenotype. Consequently, as P230p is non-essential, the *p230p* gene is the most frequently locus used to introduce additional transgenes into rodent malaria parasite genomes^15^.

The function of P230p of human malaria parasites is unknown but, like in rodent parasites, *P*. *falciparum* P230p is male specific^5,8–10,17–19^. Recently we generated transgenic *P*. *falciparum* parasites where we disrupted the *p230p* gene by introducing transgenes into this locus using adapted CRISPR/Cas9 methodology^20^. These *Pf*Δ*p230p* parasites show normal blood stage growth and are able produce gametocytes. In this study, we analysed the phenotype of the sexual stages and subsequent developmental mosquito-stages of these *Pf*Δ*p230p* parasites. We show that *P*. *falciparum* P230p has a vital role during mosquito transmission, which is in strong contrast to P230p of rodent malaria parasites. *PfΔp230p* male and female gametes retain P48/45 and P230 expression on male gametocytes. However, like *P*. *falciparum* mutants lacking P230, the capacity of *PfΔp230p* male gametes to bind to RBC is strongly reduced. In the absence of P230p expression, ookinete and oocyst development in *Anopheles stephensi* mosquitoes is almost absent. These observations identify P230p as one of a limited number of gamete-specific proteins critical for *P*. *falciparum* transmission.

## Results

### *P. falciparum* mutants lacking expression of *P230p* (*PfΔp230p-1* and *PfΔp230p-2*)

**Figure 1:**
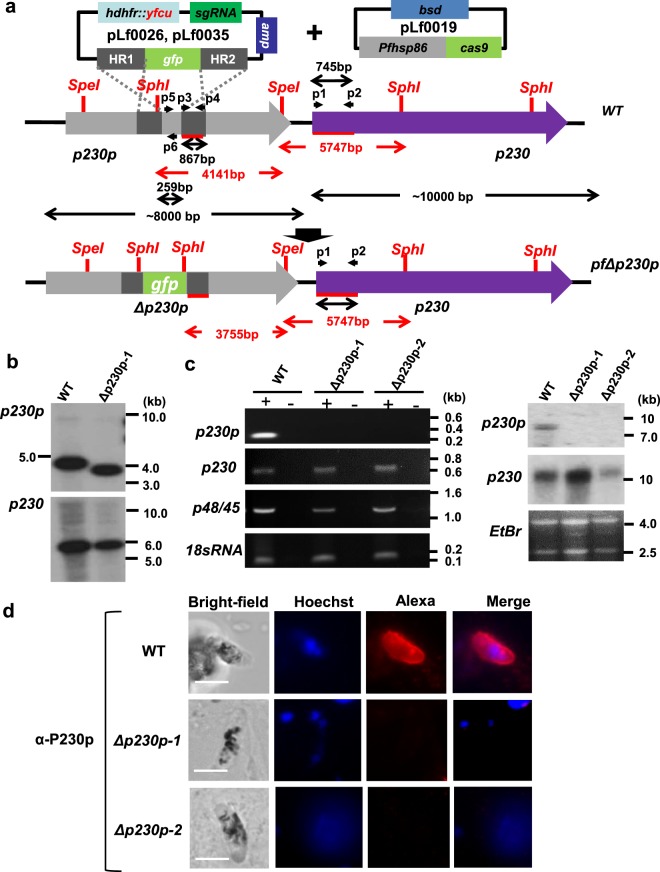
Generation and genotyping of *PfΔ230p* parasite lines and absence of *p230p* expression in *PfΔp230p* parasites. (**a**) Two *PfΔp230p* parasite lines were generated using CRISPR/Cas9 methodology as described previously^20^. The *p230p* gene was disrupted by insertion of a GFP-expression cassettes using plasmids pLf0026 (*cam* promoter driving GFP) or pLf0035 (*hsp70* promoter driving GFP). A schematic representation of the locus containing the paralogous genes *p230p* and *p230*, before and after insertion of the construct showing the location of the restriction sites (*Spe*I, *SphI*), sizes of restriction fragments (in red), location of primers (p) and the PCR amplicons and sizes of transcripts (in black) used to analyse correct disruption and transcription of the paralogous genes. (**b**,**c**) HR1, HR2: *p230p* homology regions. The figure is not shown to scale. Primer sequences can be found in Supplementary Table S1. (**b**) Southern analysis of *Sph*I/*Spe*I restricted DNA of WT and *PfΔp230p-1* parasites confirms the specific and expected disruption of the *p230p* gene locus. DNA was hybridized with a probe targeting the homology region 2 (HR2; primers p3/p4) of *p230p* (left panel) and a specific probe of 745 bp (primers p1/p2) of the 5′ *p230* open reading frame (right panel). The hybridization pattern observed with first probe identified the expected different-sized DNA fragments in WT and *PfΔp230p-1* parasites (4141 bp and 3755 bp); the second probe hybridized to a single expected fragment (5747 bp) in both WT and *PfΔp230p-1*, indicating an unaltered *p230* locus. Uncropped images of the Southerns are shown in Supplementary Fig. S3. (**c**) Transcription analysis of the 6-Cys family members *p230p*, *p230* and *p48/45* in WT and *PfΔ230p* parasites by RT-PCR and Northern blot. Left panel: RT-PCR amplified transcripts of *p230p* (primers p5/p6; expected size: 259 bp), *p230* (primers p1/p2; expected size: 745 bp), *p48/45* (primers p7/p8; expected size: 1219 bp) and *18sRNA* (primers p9/p10; expected size: 165 bp). + and − denote the presence or absence of reverse transcriptase. Uncropped images of gels are shown in Supplementary Fig. S4. Right panel: Northern blot analysis of *p230p* and *p230* transcripts confirming the absence of *p230p* and presence of *p230* transcripts in *PfΔ230p* parasites. Upper panel: hybridization with an internal probe (259 bp) from *p230p* (primers p5/p6, WT expected size: ~8 kb); middle panel hybridization with a probe against the 5′ *p230* open reading frame (primers p1/p2, expected size: ~10 kb); lower panel: ethidium bromide (*EtBr*) stained RNA as loading control. Uncropped images of the Northern blot analyses are shown in Supplementary Fig. S5. The size of expected RT-PCR products and transcripts are shown in black in (**a**). Primer sequences are shown in Supplementary Table S1. (**d**) Immunofluorescence analyses of mature, stage V, WT and PfΔp230p gametocytes. Fixed cells were labelled with mouse anti-P230p polyclonal serum (anti-rMBP.PfB0400w) and with secondary conjugated antibodies anti-IgG Alexa Fluor® 594 (red). Nuclei stained with the DNA-specific dye Hoechst-33342. All pictures were recorded with standardized exposure/gain times; anti-IgG Alexa Fluor® 594 (red). 0.6 s; Hoechst (blue) 0.136 s; bright field 0.62 s (1x gain). Scale bar, 7 µm.

We recently generated two transgenic *P*. *falciparum* mutants, where different GFP-expression cassettes had been introduced into the *p230p* gene locus using CRISPR/Cas9 technology^20^. In these mutants, GFP@cam and GFP@hsp70, GFP expression is driven by promoters of two different genes, *calmodulin* and *hsp70*. The introduction of GFP-expression cassette resulted in the disruption of the *p230p* gene including removal of 259 bp of the *p230p* coding sequence (Fig. 1a). We name here these two mutants *Pf*Δ*p230p-1* (GFP@cam) and *Pf*Δ*p230p-2* (GFP@hsp70). Correct integration of the GFP-expression cassettes in the *p230p* locus has been demonstrated by diagnostic PCR and Southern analysis^20^. The *p230p* gene is a paralogue of the *p230* gene, which is located directly down-stream of *p230p*. To show that the integration of the GFP-expression cassette disrupted only the *p230p* gene and did not alter the *p230* locus, we performed additional Southern analysis of *Sph*I/*Spe*I restricted DNA of WT and *PfΔp230p-1*, using a probe targeting the *p230p* gene-homology region (867 bp) and a probe specific for 745 bp of the *p230* open reading frame. The first probe hybridized to expected DNA fragments which differ in size in WT and *PfΔp230p-1* (4141 bp and 3755 bp, respectively), and the second probe hybridized to a fragment of the same size (5747 bp) in WT and *PfΔp230p-1* (Fig. 1a,b; see Supplementary Table S1 for primer sequences). These hybridisation results indicate the specific targeting of the *p230p* locus by the CRISPR/Cas9 constructs.

P230p is expressed exclusively in male gametocytes as has been demonstrated by immunofluorescence and PCR analyses as well as RNAseq and proteome analyses of separated *P*. *falciparum* male and female gametocytes^8–10,17–19^. In contrast, the paralog P230 and P48/45 have comparable expression levels in both males and females parasites^8–10^ (Supplementary Table S2). To demonstrate that gametocytes of *Pf*Δ*p230p-1* and *Pf*Δ*p230p-2* were deficient in *p230p* expression, we performed RT-PCR and Northern blot analysis using mRNA isolated from gametocyte cultures (Fig. 1c). No *p230p* transcripts were detected by RT-PCR in gametocytes from either *Pf*Δ*p230p-1* or *Pf*Δ*p230p-2*, whereas a *p230p* transcript (259 bp) was amplified from WT gametocytes. We were able to amplify *p230* and *p48/45* transcripts (745 and 1219 bp respectively) from gametocytes of WT and the *Pf*Δ*p230p* mutant lines. The lack of *p230p* transcripts and presence of *p230* transcripts in gametocytes of the mutant lines was confirmed by Northern blot analysis using the probes specific for the *p230p* and *p230* gene loci (Fig. 1c).

In addition, we analysed P230p expression by immunofluorescence microscopy using polyclonal antiserum against P230p^17^. This antiserum reacted only to WT gametocytes and no signal was detected in *Pf*Δ*p230p* gametocytes (Fig. 1d). Combined our analyses show correct disruption of *p230p* in *PfΔp230p* parasites resulting in absence of expression of *p230p* in gametocytes whereas the paralogous gene *p230* is transcribed. The staining pattern in WT gametocytes suggests P230p is located at the surface in the mature stage V gametocyte, and is in agreement with the localisation in *P*. *falciparum* gametocytes expressing a GFP-tagged version of P230p (P230p-GFP^9^). We also analysed P230p expression in activated male gametocytes, both using the polyclonal antiserum against P230p and in gametocytes of the transgenic P230p-GFP line. We were unable to clearly detect P230p in male gametes using the polyclonal serum or in live gametes of the P230P-GFP line but, staining with anti-GFP antibodies demonstrated that the tagged-protein was present either in or on male gametes (Supplementary Fig. S1a–c). While P230p is expressed in/on male gametes it is likely to be weakly expressed given the low GFP fluorescence and that there was no detectable signal with polyclonal serum.

### Mosquito transmission of *PfΔp230p-1* and *PfΔp230p-2* parasites is strongly reduced

**Figure 2:**
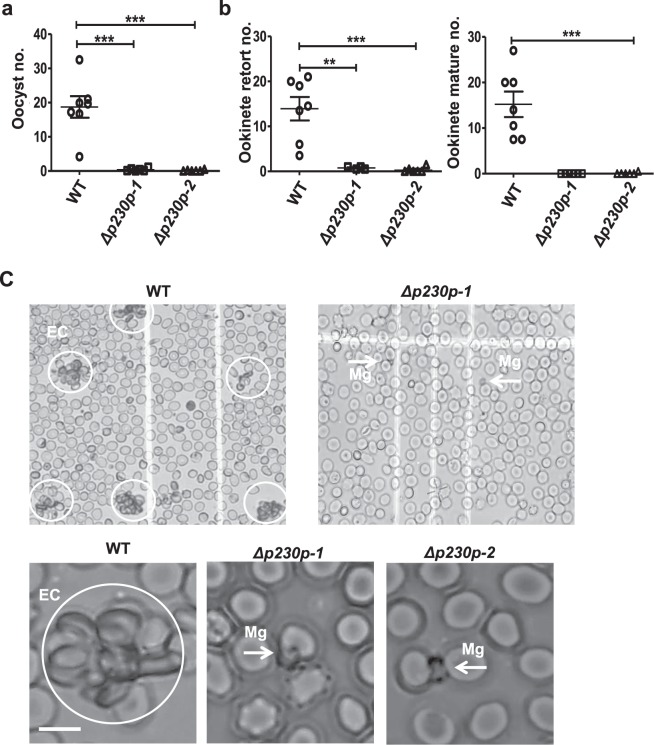
Mosquito development (ookinete and oocyst formation) and *in vitro* formation of exflagellation centres of *PfΔp230p* parasites. (**a**) Mean oocyst numbers in *A*. *stephensi* mosquitoes at day 8 after feeding in different experiments (exp.) with 10–20 mosquitoes/exp.: WT (7 exp.); *PfΔp230p-1* (6 exp.); *PfΔp230p-2* (6 exp.). ***p = 0.002 (unpaired T-test). (**b**) Mean ookinete numbers (retort and mature forms) in *A*. *stephensi* mosquitoes 24 hours after feeding. Left panel: retort (immature) ookinetes with 10–20 mosquitoes/exp.: WT (7 exp.); *PfΔp230p-1* (4 exp.); *PfΔp230p-2* (6 exp.). **p = 0.005 and ***p = 0.0006 (unpaired T-test). Right panel: mature ookinetes with 10–20 mosquitoes/exp.: WT (7 exp.); *PfΔp230p-1* (6 exp.); *PfΔp230p-2* (6 exp.). ***p = 0.0004 (unpaired T-test). (**c**) Exflagellation centres (EC, circles) as observed by light microscopy analysis of live preparations of male gametocytes between 10 and 20 min after activation examined in a Bürker cell chamber. Only WT activated male gametocytes attach to red blood cells and form characteristic exflagellation centres (left panel; white circles). See also Supplementary Videos S1–6 for the absence/presence of exflagellation centres. Arrows indicate exflagellating male gametocytes (MG) of *PfΔp230p*. Scale bar, 7 µm.

Since multiple members of the 6-cys family play a role in mosquito transmission (i.e. P48/45, P47, P230^5–7,11–14^) we analysed the ability of mosquitoes to transmit the *PfΔp230p* lines. *A*. *stephensi* mosquitoes were fed with WT and *PfΔp230p* gametocytes using the standard membrane feeding assay and the number of oocysts and salivary gland sporozoites were determined at day 6 and day 14 respectively. We fed WT and *PfΔp230p* gametocytes in independent experiments and used different clones of the mutant parasites (WT, 7 experiments (exp.); *PfΔp230p-1* clone 0022cl1, 6 exp.; *PfΔp230p-1* clone 0022cl5, 5 exp.; *PfΔp230p-2* clone 0035cl4, 6 exp). In all experiments we observed a >98% reduction in oocyst development in mosquitoes that had been fed with *PfΔp230p* parasites; mean oocyst numbers ranging from 4 to 32 for WT-infected mosquitoes compared to 0.1 to 0.7 in *PfΔp230p-*infected mosquitoes (100 mosquitoes analysed per experiment; Fig. 2a, Supplementary Table S3). In *PfΔp230p*-infected mosquitoes we observed a maximum of 5 oocysts per mosquito compared to 40 after WT feeding. No sporozoites were observed in salivary glands of *PfΔp230p*-infected mosquitoes. These results indicate the *P*. *falciparum* P230p plays an important role in mosquito transmission.

### The formation of ookinetes in *PfΔp230p-1* and *PfΔp230p-2* parasites is severely compromised

To better define the role of P230p in *P*. *falciparum* mosquito transmission we analysed gametocyte, gamete and ookinete formation of the two mutant lines. The *in vitro* production of male and female gametocytes of both *PfΔp230p-1 and PfΔp230p-2* were in the same range as WT parasites (Table 1). However, the number of ‘retort-form’ and mature ookinetes was reduced by >97% in midguts of *A*. *stephensi* mosquitoes at 22 h. after feeding (Fig. 2a, Table 1). In *PfΔp230p* infected mosquitoes we observed no or very low numbers of retorts and mature ookinetes, with mean numbers ranging from only 0 to 1.5 (retorts) and 0 to 0.5 (mature ookinetes). In WT infected mosquitoes the numbers of retorts and mature ookinetes ranged between 6–21 and 7.5–27, respectively. This strong reduction in ookinetes numbers, indicate that either fertilisation or the development of fertilised female gametes is interrupted. We cannot discriminate between these two possibilities, since *P*. *falciparum* fertilised female gametes cannot easily be distinguished from unfertilized female gametes, thereby hampering quantification of fertilisation. However, since P230p is specifically expressed in male gametocytes the most likely explanation for the reduced ookinete formation is the inability of *PfΔp230p* male gametes to fertilize females. We therefore next analysed the formation of male gametes in more detail.

**Table 1:** Gametocyte production, gamete formation (exflagellation) and ookinete formation of WT and *pfΔp230p* parasites.

Lines	Gametocyte production		No. of exflagellating males (SD)^c^	Exflagellation centers^d^	No. of retort ookinetes mean (range)^e^	No. of mature ookinetes mean (range)^f^
	Stage II % (SD)^a^	Stage V (m/f) % (SD)^b^				
***WT***						
*NF54* (*7 exp*.)	0.21 (0.06)	m: 0.18 (0.11) f: 0.41 (0.23)	9070.7 (2.68) *(3 exp.)*	+++	13.92 (6–21) *(7 exp.)*	15.21 (7.5–27)
***Δp230p-1***						
*0022cl1* (*4 exp*.)	0.15 (0.05)	m: 0.13 (0,08) f: 0.28 (0,13)	9906.0 (3,25) *(3 exp.)*	−	0.75 (0.5–1) *(4 exp.)*	0
*0022cl5* (*2 exp*.)	0.20 (0.06)	m: 0.12(0,07) f: 0.38 (0,13)	ND	ND	0.75 (0.5–1) *(2 exp.)*	0
***Δp230p-2***						
*0035cl4* (*6 exp*)	0.40 (0.15)	m: 0.06 (0,05) f: 0.36 (0,14)	4872.3 (0.75) *(3 exp.)*	−	0.33 (0–1.5) *(6 exp.)*	0.08 (0–0.5)

^a^Mean percentage of stage II gametocytes (per 100 red blood cells)in day 8 cultures in 2–7 experiments (exp.).

^b^Mean percentage of stage V male (m) and female (f) gametocytes (per 100 red blood cells) in day 14 cultures in 2–7 experiments (exp.).

^c^Mean number of exflagellating male gametocytes (per 1 × 10^5^ red blood cells) at 10–20 min after activation of day 14 cultures (s.d.: standard deviation).

^d^Level of the formation of exflagellation centers: +++: >90% of exflagellating males form exflagellation centers; − <1% of the exflagellating males form exflagellation centers.

^e^Mean number of retort form ookinetes in a pool of 5 mosquitoes at day 22 after feeding. Range corresponds to the mean number of retorts in multiple experiments (5–7 exp. per line; 10–20 mosquitoes per exp.).

^f^Mean number of mature ookinetes in a pool of 5 mosquitoes at day 22 after feeding. Range corresponds to the mean number of ookinetes in multiple experiments (5–7 exp. per line; 10–20 mosquitoes per exp.).

### *PfΔp230p-1* and *PfΔp230p-2* male gametes are unable to generate exflagellation centres

**Figure 3:**
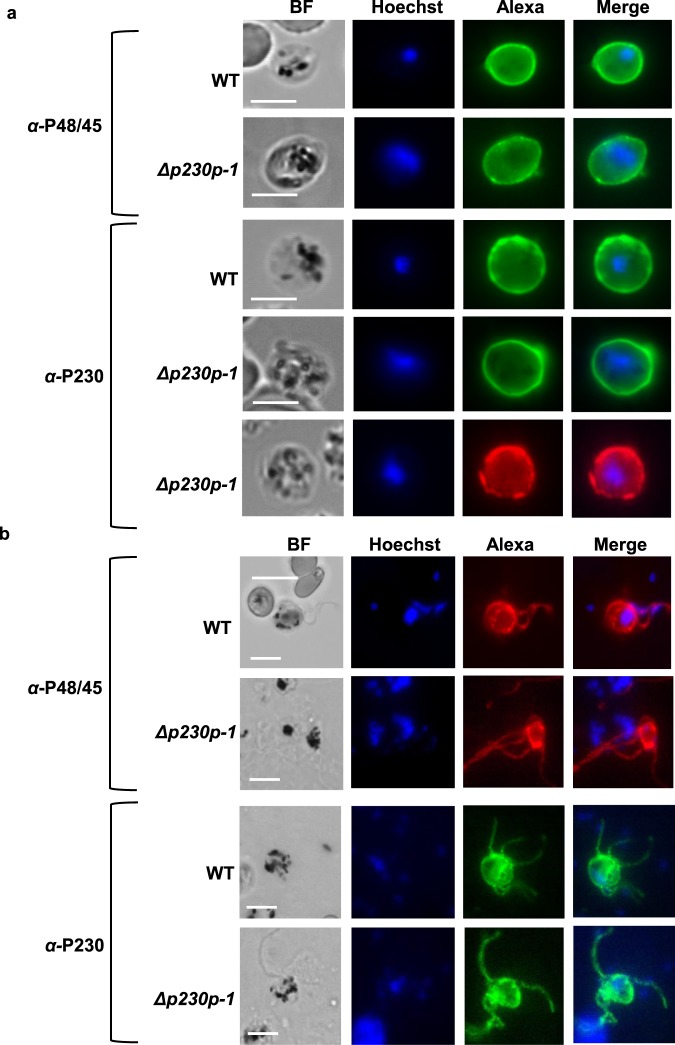
Expression of P230 an P48/45 in activated female and male gametes of *PfΔp230p-1*. (**a**) Immunofluorescence analyses of female gametes 30 minutes after gametocyte activation in fetal calf serum. Unfixed parasites were labelled with mouse anti-P230 (MAb 63F2A2) or rat anti-P48/45 (MAb 85RF45.1) antibodies followed by secondary conjugated antibodies (i.e. anti-rat, anti-mouse IgG Alexa Fluor® 488 (green) or anti- mouse IgG Alexa Fluor ® 594 (red)). Nuclei stained with the DNA-specific dye Hoechst-33342. All pictures were recorded with standardized exposure/gain times; Alexa Fluor® 488 (green) 0.7 s; anti-IgG Alexa Fluor ® 594 (red) 0.6 s; Hoechst (blue) 0.136 s; bright field 0.62 s (1x gain). Scale bar, 7 µm. (**b**) Immunofluorescence analyses of male gametes 15 minutes after gametocyte activation in fetal calf serum. Cells were fixed with methanol and labelled with mouse anti-P230 (MAb 63F2A2) or rat anti-P48/45 (MAb 85RF45.1) antibodies followed by secondary conjugated antibodies (i.e. anti-mouse IgG Alexa Fluor® 488 (green) or anti-rat IgG Alexa Fluor® 594 (red)). Nuclei stained with the DNA-specific dye Hoechst-33342. All pictures were recorded with standardized exposure/gain times; Alexa Fluor ® 488 (green) 0.7 s; anti-IgG Alexa Fluor ® 594 (red). 0.6 s; Hoechst (blue) 0.136 s; bright field 0.62 s (1x gain). Scale bar, 7 µm.

Both gametocyte production and sex ratio of mature gametocytes at day 14 were comparable between WT and *PfΔp230p* parasites (Table 1) and morphologically, at the light microscopy level, there is no difference between WT and *PfΔp230p* gametocytes (data not shown). After activation in FCS mature gametocytes from both WT and *PfΔp230p* cultures readily formed high numbers of exflagellating male gametocytes as observed by light-microscopy. We estimate that >90% of stage V male *PfΔp230p* gametocytes showed exflagellation (from 3 experiments) (Table 1). These observations indicate that the formation of male gametes is not affected by the absence of P230p. However, a striking difference was the absence of *PfΔp230p* male gamete attachment to uninfected RBC and the formation of exflagellation centres observed 15–30 min post activation (Fig. 2b and Supplementary Videos S1–S6). Such exflagellation centres generally consist of one or more exflagellating male gametes attaching to a number of RBC^21^. While WT stage V gametocytes formed such centres by more than 90% of the activated male gametocytes, this was observed in less than 5% of activated *PfΔp230p* males (Table 1). These results indicate that in the absence of P230p, male gametes were incapable of effectively attaching to RBC. This phenotype is very similar to the phenotype described for *P*. *falciparum* mutants lacking P230 and indicates that both P230 and P230p play a role in interactions of male gametes with RBC. Whether the inability of male gametes to interact with RBC is solely responsible for the reduced ookinete formation, or whether P230p has an additional role in fertilisation, is unknown. It has been shown that the 6-cys family members, P230 and P48/45, form complexes with other proteins on the surface of gametes^22,23^. We therefore examined if the expression of P230 and P48/45 was altered in activated *PfΔp230p-1 and PfΔp230p-2* gametocytes by immunofluorescence analysis. Using anti-P230 and P48/45 antibodies we demonstrated that P230 and P48/45 were present in both activated *PfΔp230p* female and male gametocytes/gametes where staining patterns were comparable to what was observed in activated WT gametocytes (Fig. 3a,b and Supplementary S1d). The combined results indicate that the formation of exflagellation centres and subsequent reduced ookinete formation is directly P230p dependent and not a consequence of the loss of P230 or P48/45 on gametes in the *PfΔp230p* gametes.

The role of P230 and P230p in *P*. *falciparum* gamete binding to RBC is different to the role of these proteins in the rodent parasite *P*. *berghei*. Single gene-deletion mutants lacking expression of either P230 or P230p in *P*. *berghei* exhibit formation of exflagellation centres like WT, indicating that male gametes of these mutants bind normally to RBC^5^. To examine a possible compensatory role in RBC binding of the two *P*. *berghei* paralogs we generated a *p230* and *p230p* double gene deletion mutant (Supplementary Fig. S2a,b). Activated male gametocytes of this mutant, *PbΔp230Δp230p*, formed WT-like levels of exflagellation centres (Supplementary Fig. S2c, Supplementary Videos S7–12, Supplementary Table S4) demonstrating an absence of a role of these proteins in RBC binding of *P*. *berghei* male gametes.

**Figure 4:**
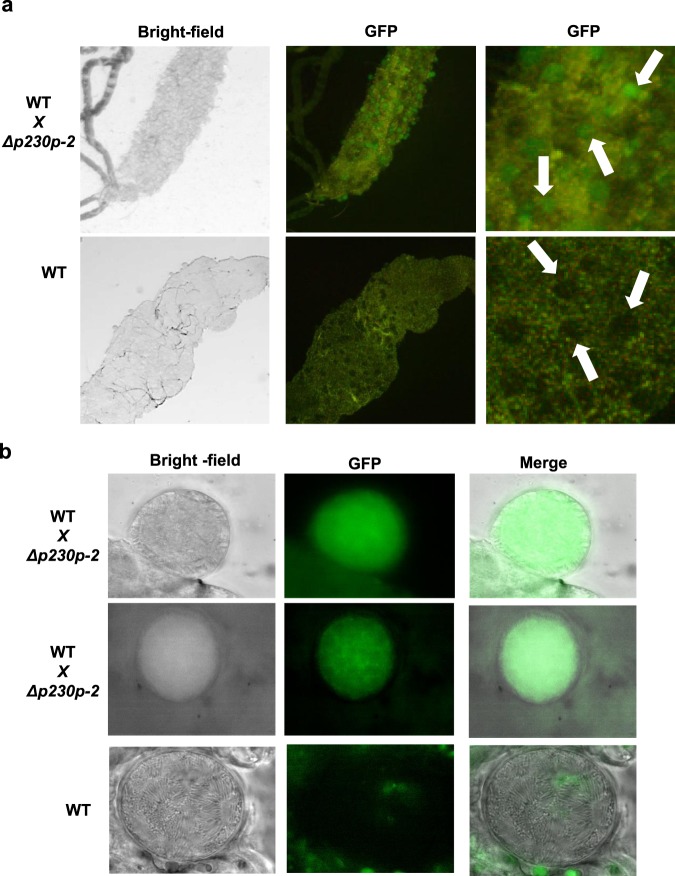
Crossing of GFP-expressing *PfΔp230p* gametocytes with WT gametocytes results in the formation of GFP-positive oocysts. (**a**) GFP-positive oocyst in midguts of *A*. *stephensi* mosquitoes fed on a mixture of *PfΔp230p-2* and *WT* gametocytes (day 10 after feeding). Arrows indicate GFP-positive oocysts in the WT and *PfΔp230p-2* cross and GFP-negative oocysts in WT fed mosquitoes. (**b**) GFP-positive and GFP-negative oocysts in mosquitoes fed on a mixture of *PfΔp230p-2* and WT gametocytes or only WT gametocytes (day 10 after feeding). See Table 25 for the ratio of GFP-positive and GFP-negative oocysts in mosquitoes fed on a mixture of *PfΔp230p-2* and *WT* gametocytes. All pictures were recorded with standardized exposure/gain times to visualize differences in fluorescence intensity (GFP 0.7 s; bright field 0.62 s (1x gain)).

Finally, we examined fertility of female gametes of the *PfΔp230p* lines, by crossing the GFP-expressing *PfΔp230p* gametocytes with WT gametocytes and examining parasite development in mosquitoes. Mosquitoes with both GFP-positive and GFP-negative oocysts were obtained in multiple experiments (Fig. 4; Table 2). GFP-positive oocysts can only result from cross-fertilisation of WT gametes and *PfΔp230p* gametes. In view of the male-specific expression of P230p and the male phenotype of *PfΔp230p* parasites, the presence of the GFP-positive oocysts most likely result from cross-fertilisation between WT male gametes and *PfΔp230p* female gametes. These observations are in support of normal fertility of *PfΔp230p* female gametes and reduced fertility of *PfΔp230p* male gametes.

**Table 2:** GFP-positive and GFP-negative oocysts after crossing *P*. *falciparum* WT and *pfΔp230p* gametocytes.

Parasites	Gametocyte production	No. of exflagellating males^b^	Ratio WT/*Δp230p* gams in cross^c^	No. of oocyst Mean (range)^d^	GFP positive oocyst (%)^e^
	Stage V (m/f)%(SD)^a^				
***Cross A:***					
*WT*	m: 0.5 (0.5)	0.5	1/1 *(1 exp.)*	13 *(1 exp.)*	0
	f: 1.3 (1.2)				
*Δp230p-2*	m: 0.1 (0.4)	0.1			
	f: 0.2 (1.2)				
***Cross B:***					
*WT*	m: 0.5 (0.8)	0.5	1/2 *(2 exp.)*	40 (50–60) *(2 exp.)*	58%
	f: 1.0 (0.8)				
*Δp230p-2*	m: 0.9 (0.6)	0.8			
	f: 1.8 (0.8)				
***Cross C:***					
*WT*	m: 0.5 (0.8)	0.2	1/3 *(1 exp.)*	59 *(1 exp.)*	41%
	f: 1.0 (0.8)				
*Δp230p-2*	m: 0.6 (0.7)	0.3			
	f: 1.1 (0.4)				

^a^Mean percentage of stage V male (m) and female (f) gametocytes (per 100 red blood cells) in day 14 cultures in 1–2 experiments (exp.). SD: Standard deviation.

^b^Mean number of exflagellating male gametocytes (per mL of culture) at 10–20 min after activation of day 14 gametocyte cultures (SD: standard deviation).

^c^WT and *PfΔp230p* gametocytes were mixed in different ratios (1:1, 1:2, and 1:3) based on exflagellating male gametocytes counts per ml of gametocyte culture after activation with FCS.

^d^Mean number of oocysts per mosquito at day 8 after feeding. Range corresponds to the mean number of oocyst in different experiments (1–2 exp. per crossing; 10–30 mosquitoes per exp.).

^e^Percentage of GFP positive oocyst analysed in 5 individuals mosquitoes (1–2 exp.).

## Discussion

We demonstrate that *P*. *falciparum* P230p plays a vital role in parasite transmission through mosquitoes. Mutants lacking expression of P230p (*PfΔp230p*) have a strong reduction (>98%) in ookinete formation, which in turn results in a strong reduction in oocyst formation and absence of sporozoites in salivary glands. We show that the *PfΔp230p* male gametes have lost the capacity to bind to RBC and could not form the characteristic exflagellation centres. A function of P230p in male gamete fertility is in agreement with male specific expression of *Pf*P230p and concomitant absence in female gametocytes/gametes^8–10,17–19^. Indeed the results of crossing experiments in mosquitoes, performed using a mixture of WT and *PfΔp230p* gametocytes, indicate that *PfΔp230p* females retain their fertility. The important role that *P*. *falciparum* P230p plays in mosquito transmission does not match the redundant function of P230p in the rodent parasites *P*. *berghei* and *P*. *yoelii*. Rodent parasites also express P230p specifically in male gametocytes^24^, but mutants lacking P230p have no discernible defect and exhibit normal gametocyte/gamete formation, are fully able to form exflagellation centres and mosquito transmission is similar to WT parasites^5,15^. We also demonstrate that the *P*. *berghei* P230 is not compensating for the loss of its paralogue P230p, since activated male gametocytes of *P*. *berghei* mutants lacking expression of both P230 and P230p can still bind to RBCs and form exflagellation centres. These observations demonstrate a critical difference in the function P230p performs in rodent and human malaria parasites. For a few other 6-Cys proteins there has been evidence for functional differences between the orthologs of rodent and human parasites. For example, the female-specific P47 protein is vital for the fertility of *P*. *berghei* female gametes, while this protein appears not to be crucial for *P*. *falciparum* female gamete fertility^5,11^. Analysis of *P*. *berghei* mutants lacking P45/48 and P230 demonstrates that these proteins are male-specific fertility factors^5,6^. In contrast, *P*. *falciparum* P48/45 and P230 are expressed in both males and female gametes^8–10^, which may suggest a role for these proteins in both male and female gamete fertility. Also other proteins expressed in gametocytes/gametes functional differences have been demonstrated between the equivalent proteins in rodent and human malaria parasites, for example members of the LCCL protein family. In rodent parasites most LCCL members are expressed after fertilisation, in the ookinete stage, and play a role in sporozoite formation^25,26^, whereas in *P. falciparum* these proteins are expressed in gametocytes and are part of protein complexes on the surface of gametocytes/gametes^27,28^.

The lack of RBC binding of *PfΔp230p* male gametes is similar to the phenotype of *P*. *falciparum* mutants lacking expression of its paralog, P230^7^. These observations suggest that both proteins have a similar, but not interchangeable, function in RBC binding. We provide evidence that *PfΔp230p* male and female gametes retain expression of both P230 and P48/45, indicating that the lack of RBC binding is not due to the absence of expression of P230 and/or P48/45. These observations would suggest that RBC binding of male gametes is not due to direct interactions of P230 to RBC receptors as was also demonstrated in the studies using males lacking expression of P230^7^. Moreover, P230 unlike P230p is also expressed at the surface of female gametes^8–10,23^, which makes it less likely that P230 interacts directly with RBC.

P230 and P48 form complexes with several other proteins at the surface of female gametes and zygotes^23,29,30^. Given that of P48/45, P230 and P230p are expressed in male gametes, it is conceivable that comparable complexes that may include additional proteins, are also formed at the surface of male gametes. The absence of either P230 or P230p may affect the correct formation of such protein complexes at the gamete surface, which may in turn lead to the same loss of RBC binding phenotype observed in mutants that lack either paralog. This would indicate that neither P230 nor P230p but rather other parasite proteins/factors are directly responsible for binding to RBC receptors. Future studies are needed to unravel in more detail the molecular interactions between male gametes and RBC and the *Plasmodium* ligand(s) that bind to the putative proteins, sialic acid and/or glycophorin receptors on the RBC surface^21^.

Whether the reduction in fertilisation and ookinete formation within the mosquito midgut of parasites lacking either P230 or P230p is directly due to the inability of male gametes to bind to RBC or whether these proteins have an additional role in fertilisation remains unknown. Studies on male gametes lacking P230 showed that the inability to form exflagellation centres did not affect the release of male gametes from activated gametocytes^7^ and we also observed *in vitro* that *PfΔp230p* male gametes were released after gametocyte activation. It has been suggested that the RBC interactions may trigger changes in the gamete that are required for fertilisation such as the release of additional proteins, which through a process analogous to sperm capacitation, permit the male gamete to be able to bind to molecules in the zona pellucida of the oocyte and thereby initiating the process of male penetration of the female gamete^21^. Unfortunately, efficient *in vitro* assays for *P*. *falciparum* fertilisation are absent^31^ and fertilised female gametes cannot easily be distinguished from unfertilized female gametes, thereby hampering more detailed analyses and quantification of fertilisation events.

Our study expands the role of the 6-Cys proteins in fertilisation and specifically demonstrates that P230p, like P230 and P48/45, has a clear and vital role in *P*. *falciparum* male fertility, zygote formation and parasite transmission through mosquitoes.

## Materials and Methods

### Parasites and culture

We analysed wild type (WT) *P*. *falciparum* NF54 parasites and two mutant lines *PfΔp230p-1* (GFP@cam clones 0022cl1 and 0022cl5) and *PfΔp230p-2* (GFP@hsp70 clone 0035cl4) with a disrupted *p230p* gene locus (PF3D7_0208900). These mutants were generated in NF54 parasites by introducing a GFP-reporter cassette into the *p230p* gene locus using CRISPR/Cas9 methodology^20^. In the two mutants, GFP is either under the control of the promoter from *calmodulin* (*cam*; PF3D7_1434200) or from *heat shock protein 70* (*hsp70*; PF3D7_0818900). The genotype and phenotype of (asexual) blood stages of these mutants have been reported previously^20^. WT NF54 parasites^32^ had been obtained from the Radboud University Medical Center (Nijmegen, The Netherlands). NF54 parasites were cultured following the standard conditions in RPMI-1640 culture medium supplemented with L-Glutamine 25 mM HEPES (Gibco Life Technologies) and 50 mg/L hypoxanthine (Sigma). Culture medium was supplemented with 10% human serum and 0.225% NaHCO_3_. Parasites were cultured at a 5% hematocrit under 4% O_2_, 3% CO_2_ and 93% N_2_ gas-conditions at 75 rpm at 37 °C in a semi-automated culture system^33^. Fresh human serum and human red blood cells (RBC) were obtained from the Dutch National Blood Bank (Sanquin Amsterdam, the Netherlands; permission granted from donors for the use of blood products for malaria research and microbiology safety-tests).

In addition, a *P*. *falciparum* (3D7) transgenic line that expresses a GFP-tagged version of *Pfp230p* (*Pfp230p-*GFP) was analysed for P230p expression and localisation. This line has been engineered to express endogenous PfP230p fused to GFP to its carboxyl terminal and was generated using a single cross-over recombination strategy^9^.

Two different *P*. *berghei* ANKA mutants were analysed that have been previously generated. One with a *p230p* gene disruption (line 676m1cl1; *PbΔp230p*; RMgm-29; www.pberghei.eu)^34^ and the other with a *p230* gene disruption (line 310cl1; *PbΔp230*; RMgm-350; www.pberghei.eu)^5^. In addition, we generated a double gene deletion *P*. *berghei* ANKA mutant with both the *p230p* (PBANKA_0306000) and *p230* (PBANKA_0306100) gene loci disrupted (see below).

### Animal ethics statement

Female OF1 mice (6–8 weeks old; Charles River/Janvier) were used. All animal experiments of this study were in accordance with relevant guidelines and regulations approved by the Animal Experiments Committee of the Leiden University Medical Center (DEC 12042). The Dutch Experiments on Animal Act is established under European guidelines (EU directive no. 86/609/EEC regarding the Protection of Animals used for Experimental and Other Scientific Purposes).

### Generation of the *P. berghei* double knock-out mutant *PbΔp230Δp230p*

To generate a *P*. *berghei* mutant lacking expression of both P230 and P230p we disrupted the *p230* locus in the existing *PbΔp230p* mutant (676m1cl1; see above) which has a disrupted *p230p* locus. To disrupt *p230* we used a DNA construct that had been used to create the mutant *PbΔp230* (310cl1; see above). This construct (pL1139) integrates by double cross integration and replaces (part of) the *p230* locus with the selectable marker cassette containing *Toxoplasma gondii* dihydrofolate reductase/thimidylate synthase (*tgdhfr/ts*)^5^. Parasites of line 676cl1 were transfected with this construct (exp. 2764) using standard transfection technologies and selection with pyrimethamine^35^. Selected parasites were cloned by limiting dilution and mutant 2764cl3 was used for genotype and phenotype analysis.

### Genotyping and phenotyping *P. berghei* mutant *PbΔp230Δp230p*

Correct disruption of the *p230p* and *p230* gene loci was performed by diagnostic PCR-analysis and Southern analysis of pulsed field gel (PFG) separated chromosomes as described previously^35^. Briefly, for the PCR-analysis confirmation of disruption of *p230* was performed using the primers p13/p14 for 5′ integration and p17/p18 for 3′ integration of the construct and for *p230p*, 5′ and 3′ integration with primer pairs p21/p22 and p25/p26, respectively (see Supplementary Fig. S2 and Supplementary Table S1 for details of the primers and the PCR fragments). For Southern analysis, diagnostic probes against the 3′UTR of *pbdhfr/ts* and the *tgdhfr/ts* selectable marker were used.

*In vitro* activation of gametocytes to determine exflagellation, formation of exflagellation centres and formation of ookinetes were performed as described^5,35^. In brief, 10–20 µl of tail blood from infected mice containing gametocytes was diluted in 1 ml of activation medium. Within 12–20 min after activation, exflagellating male gametocytes and exflagellation centres were quantified in a Bürker cell counter and 18–24 h later the number of zygotes/ookinetes formed was quantified.

### Genotyping *P. falciparum* mutants *PfΔp230p-1* and *PfΔp230p-2*

Supplementary TablesSouthern analysis of restricted genomic DNA to confirm disruption of *p230p* in mutants *PfΔp230p-1* and *PfΔp230p-2* have been reported^20^. We performed additional Southern analysis to confirm disruption of *p230p* and to confirm that the neighbouring *p230* gene locus (PF3D7_0209000) remained unmodified. Total DNA was isolated from infected red blood cells (iRBC) obtained from 10 ml cultures (parasitemia 5–10%, 5% hematocrit), pelleted by centrifugation (400 *g*; 5 min). RBC were lysed with 5–10 ml of cold (4 °C) erythrocyte lysis buffer (10x stock solution 1.5 M NH_4_Cl, 0.1 M KHCO_3_, 0.01 M Na_2_EDTA; pH 7.4^35^; and parasites pelleted by centrifugation (400 *g* during 5 min) and treated with RNAse and proteinase-K before DNA isolation by standard phenol-chloroform methods. Genomic DNA was digested with *Spe*I and *Sph*I restriction enzymes (4 h at 37 °C) to confirm the specific disruption of *Pfp230p* locus. Restricted DNA was hybridized with 2 probes: one targeting the *p230p* homology region 2 (HR2) and one targeting the 5′ *p230* open reading frame (5′*-p230*) amplified from WT NF54 genomic DNA by PCR using the primers P3/P4 for HR2 and P1/P2 for 5′*-p230*, respectively (see Supplementary Table S1 for details of the primers).

### Transcriptional analyses of 6-cys family proteins in the mutants *PfΔp230p-1* and *PfΔp230p-2*

To analyse transcription of 6-cys family proteins *P*. *falciparum* gametocytes were generated using standard culture conditions (see above) with some modifications^33^. Briefly, parasites from asexual stage cultures were diluted to a final parasitemia of 0.5% and cultures were followed during 14 days without refreshing RBC. After 9 days these cultures were treated with 50 mM of N-acetyl-D-glucosamine (Sigma) to kill asexual stages and to enrich for gametocytes. At day 14 the cultures were harvested and infected RBC (iRBC), enriched for gametocytes, pelleted by centrifugation (400 *g* during 5 min), washed three times with 1X PBS and the iRBC lysed with saponin following standard procedures^36^. Total RNA was isolated from the pelleted parasites using the Kit RNA Pure Link ^TM^ RNA Mini kit (Invitrogen) according to the manufactures instructions. Northern blot analysis on the isolated RNA, was performed as previously described^36^ using probes amplified from genomic DNA from WT NF54 parasites; one targeting an internal fragment (259 bp) of *p230p* with primers P5/P6 and the other targeting a fragment (754 bp) of the 5′ *p230* open reading frame with primers P1/P2 (see Supplementary Table S1 for details of the primers). RNA (1–5 µg) isolated from the iRBC was further purified for RT-PCR analysis by adding 1X DNase I digestion buffer (Promega), 20 U of RNase inhibitor (RNasin, Promega) and 20 U of DNase I (Promega); this was incubated for 45 min at 37 °C followed by chloroform/isoamyl alcohol purification and RNA precipitated in absolute ethanol^36^ Subsequently, RT-PCR was performed using standard methods^36^. Briefly, 1–3 µg of RNA was collected for first strand cDNA synthesis using the kit SuperScript III (Invitrogen) and PCR amplification (annealing temperatures ranging 50–57 °C) was performed with KOD polymerase (Invitrogen). For amplification of the *Pf48/45* gene (PF3D7_1346700) the primers P7/P8 were used, for *Pfp230* primers P1/P2, for *Pfp230p* primers P5/P6 and for *18 S rRNA* primers P9/P10 (see Supplementary Table S1 for details of the primers).

### Expression analysis of 6-cys family proteins in *P. falciparum* gametocyes by immunofluorescence assay (IFA)

To analyse the expression of *Pf*P48/45 and *Pf*P230 in live gametocytes by immunofluorescence microscopy, 500 µl of the gametocyte culture was pelleted (400 *g* 30 s) and gametocytes activated in 1 ml of fetal calf serum (FCS) for 1 h at room temperature and samples collected for live fluorescence microscopy. To analyse *Pf*P48/45 and *Pf*P230 expression in fixed (male) gametocytes, 20 µl of the activated cells were collected 15–20 min after activation. This gamete enriched solution was placed on a microscope slide, dried for 10 min, and fixed with ice-cold methanol for 5 min. After fixation the slides were blocked with 10% of FCS in 1X PBS for 1 h. Live and fixed cells were washed with 1X PBS and incubated with monoclonal antibodies against *Pf*P48/45 (rat MAb 85RF45.1; 1:200 dilution of 5 µg/ml stock solution^37^), PfP230 (mouse MAb 63F2A2; 1:200 dilution of 5 µg/ml stock solution^38^) for 30 min at 4 °C for live imaging and 1 h at room temperature for fixed slides. Subsequently, cells were rinsed 3 times with 1X PBS and incubated with the secondary antibodies Alexa FLuor®488/594-conjugated chicken anti-rat and anti-mouse (Invitrogen Detection technologies), respectively (both at 1:200). Finally, the cells were stained with the DNA-specific dye Hoechst-33342 at a final concentration of 10 µM. Fixed cells were covered with 1–2 drops of an anti-fading agent (Vectashield), and a coverslip placed onto the cells and sealed with nail polish. Stained cells (live and fixed) were analysed for fluorescence using a Leica fluorescence MDR microscope (100x magnification). Pictures were recorded with a DC500 digital camera microscope using Leica LAS X software with the following exposure times: Alexa: 0.7 s; Hoechst 0.136 s; bright field 0.62 s (1x gain). To analyse *Pf*P230p expression in stage V gametocytes by immunofluorescence slides for microscopy analysis were prepared as follows: 20 µl of the cell suspension containing activated gametocytes was placed per well of a 8-well black cell-line diagnostic microscope slide (Thermo Scientific), that was air dried, fixed with ice-cold absolute methanol (2 min) and subsequently washed 3 times with 1X PBS. Cells were permeabilized with 0.5% of Triton X-100 in 1X PBS for 1 h and blocked with 10% FCS in 1X PBS. 20 µl of polyclonal serum raised in mice against recombinant *Pf*P230p (1:200; anti-rMBP.PfB0400w 1:200 dilution^17^) was incubated with the fixed gametocytes for 1 h at room temperature and slides were washed 3 times with 1XPBS. Subsequently each well was incubated with 20 µl goat-anti-mouse secondary IgG monoclonal antibody conjugated to Alexa FLuor®594- (Supplier; 1:200 dilution) for 1 h at room temperature. Slides were then washed 3 times with 1X PBS and stained with 20 µl of Hoechst-33342 in 1XPBS (10 µM) for 30 min at 37 °C. The slides were washed 3 times in 1X PBS and the cells were analysed for fluorescence using a Leica fluorescence MDR microscope (see above for details).

Further analysis of *Pf*P230p expression was performed by detecting the GFP pattern in the live or fixed gametocytes of *Pfp230p-*GFP line. Rabbit anti-GFP IgG (Invitrogen; 4 µg/ml) and Goat anti-rabbit IgG conjugated to Alexa FLuor®488 (Invitrogen; 4 µg/ml) were used for detection of GFP in fixed activated mature gametocytes.

### Phenotype analysis of gametocytes/gametes and mosquito stages of mutants *PfΔp230p-1* and *PfΔp230p-2*

Gametocyte development was analysed in gametocyte cultures, established as described above. Exflagellation was determined after activation of *P*. *falciparum* stage V gametocytes with FCS. To activate gametocytes 20 µl of the gametocyte cultures at day 14 were diluted 1:1 with FCS at room temperature. Gametes and exflagellation centres were examined and quantified 10–20 min after activation using a Bürker cell counter.

The number of male gametocytes per 10^5^ red blood cells (RBC) was determined in stage V gametocyte cultures by analysing Giemsa stained slides. Quantification of exflagellating males of these cultures was performed in triplicate, using a Bürker chamber (at 40X magnification). The number of exflagellating males is given as the number of exflagellating males observed per 1 × 10^5^ of total red blood cells (RBC).

Exflagellation center formation was determined by counting the number exflagellating males adhering to multiple red blood cells and forming characteristic dense clusters of RBC. Exflagellating males, which did not adhere to RBC and failed to form a characteristic dense cluster of RBC were scored as ‘non-adhering’ males. +++ denotes that more than 90% of the exflagellating males formed the dense RBC clusters and − denotes that less than 1% of exflagellating males formed the dense RBC clusters. For analysis of mosquito stages (ookinetes, oocysts and sporozoites) *A*. *stephensi* were infected using the standard membrane feeding assay (SMFA)^39,40^. Ookinetes were analysed and counted 22 h after feeding. Oocyst and salivary gland sporozoites were counted at day 6 and day 14 post feeding, respectively. For counting sporozoites, salivary glands from 10 mosquitoes were dissected and homogenized in a homemade glass grinder in 100 µl of RPMI-1640 (pH 7.2) and sporozoites were analysed in a Bürker cell counter using phase-contrast microscopy.

Cross-fertilisation of WT and *PfΔp230p* gametocytes was performed by mixing gametocytes obtained from enriched gametocyte cultures (see above) from WT and *PfΔp230p* and feeding these mixtures to mosquitoes using SMFA. In different experiments WT and *PfΔp230p* gametocytes were mixed in different ratios (1:1, 1:2, and 1:3) based on exflagellating male gametocytes counts per ml of gametocyte culture after activation with FCS. At day 10 after feeding oocyst development was analysed with a fluorescence stereomicroscope Leica MZ16 FA and GFP-fluorescence was visualized using GFP filter settings (GFP exposure time: 4,2 s). Pictures were recorded using a DM2500 digital camera.

### Statistics

All data were analyzed using the GraphPad Prism software package 5.04 (GraphPad Software, Inc). To calculate significant levels for ookinete and oocyst numbers the unpaired Student’s *t*-test was used.

## Electronic supplementary material

Video S1

Video S2

Video S3

Video S4

Video S5

Video S6

Video S7

Video S8

Video S9

Video S10

Video S11

Video S12

Supplementary information
