# Author Correction: The *Plasmodium falciparum* male gametocyte protein P230p, a paralog of P230, is vital for ookinete formation and mosquito transmission

**DOI:** 10.1038/s41598-019-43505-y

**Published:** 2019-05-03

**Authors:** Catherin Marin-Mogollon, Marga van de Vegte-Bolmer, Geert-Jan van Gemert, Fiona J. A. van Pul, Jai Ramesar, Ahmad Syibli Othman, Hans Kroeze, Jun Miao, Liwang Cui, Kim C. Williamson, Robert W. Sauerwein, Chris J. Janse, Shahid M. Khan

**Affiliations:** 1 Leiden Malaria Research Group, Parasitology, Leiden University Medical Center (LUMC), Leiden, The Netherlands; 2 Department of Medical Microbiology, Radboud University Medical Center, Nijmegen, The Netherlands; 3 Faculty of Health Sciences, Universiti Sultan Zainal Abidin, Terengganu, Malaysia; 4 Department of Entomology, The Pennsylvania State University, University Park, Pennsylvania, United States; 5 Microbiology and Immunology Department, Uniformed Services University of the Health Sciences, Bethesda, Maryland United States

Correction to: *Scientific Reports* 10.1038/s41598-018-33236-x, published online 08 October 2018.

In the Supplementary Information file originally published with this Article, Supplementary Tables S1 to S4 were omitted. This error has now been corrected in the Supplementary Information that now accompanies the Article.

