# Supplementary material for: The Plasmodium falciparum male gametocyte protein P230p, a paralog of P230, is vital for ookinete formation and mosquito transmission
Source: Sci Rep. 2018 Oct 8;8:14902. doi: 10.1038/s41598-018-33236-x (PMC6175877; doi:10.1038/s41598-018-33236-x)
Supplement: Supplementary file 13 — Supplementary information [file 41598_2018_33236_MOESM13_ESM.pdf]

Supporting information

The *Plasmodium falciparum* male gametocyte protein P230p, a paralog of P230, is vital for ookinete formation and mosquito transmission

Authors

Catherin Marin-Mogollon[*], Marga van de Vegte-Bolmer[**], Geert-Jan van Gemert[**], Fiona J. A. van Pul[*], Jai Ramesar[*], Ahmad Syibli Othman[*,***], Hans Kroeze[*], Jun Miao[****], Liwang Cui[****], Kim C. Williamson[*****] Robert W. Sauerwein[**], Chris J. Janse[*], Shahid M. Khan[*]*

Addresses

[*]Leiden Malaria Research Group, Parasitology, Leiden University Medical Center (LUMC), Leiden, The Netherlands

[**] Department of Medical Microbiology, Radboud University Medical Center, Nijmegen, The Netherlands

[***] Faculty of Health Sciences, Universiti Sultan Zainal Abidin, Terengganu, Malaysia

[****]Department of Entomology, The Pennsylvania State University, University Park, Pennsylvania, United States.

[*****]Microbiology and Immunology Department, Uniformed Services University of the Health Sciences, Bethesda, MD, United States.

*Correspondence to S.M.Khan@lumc.nl

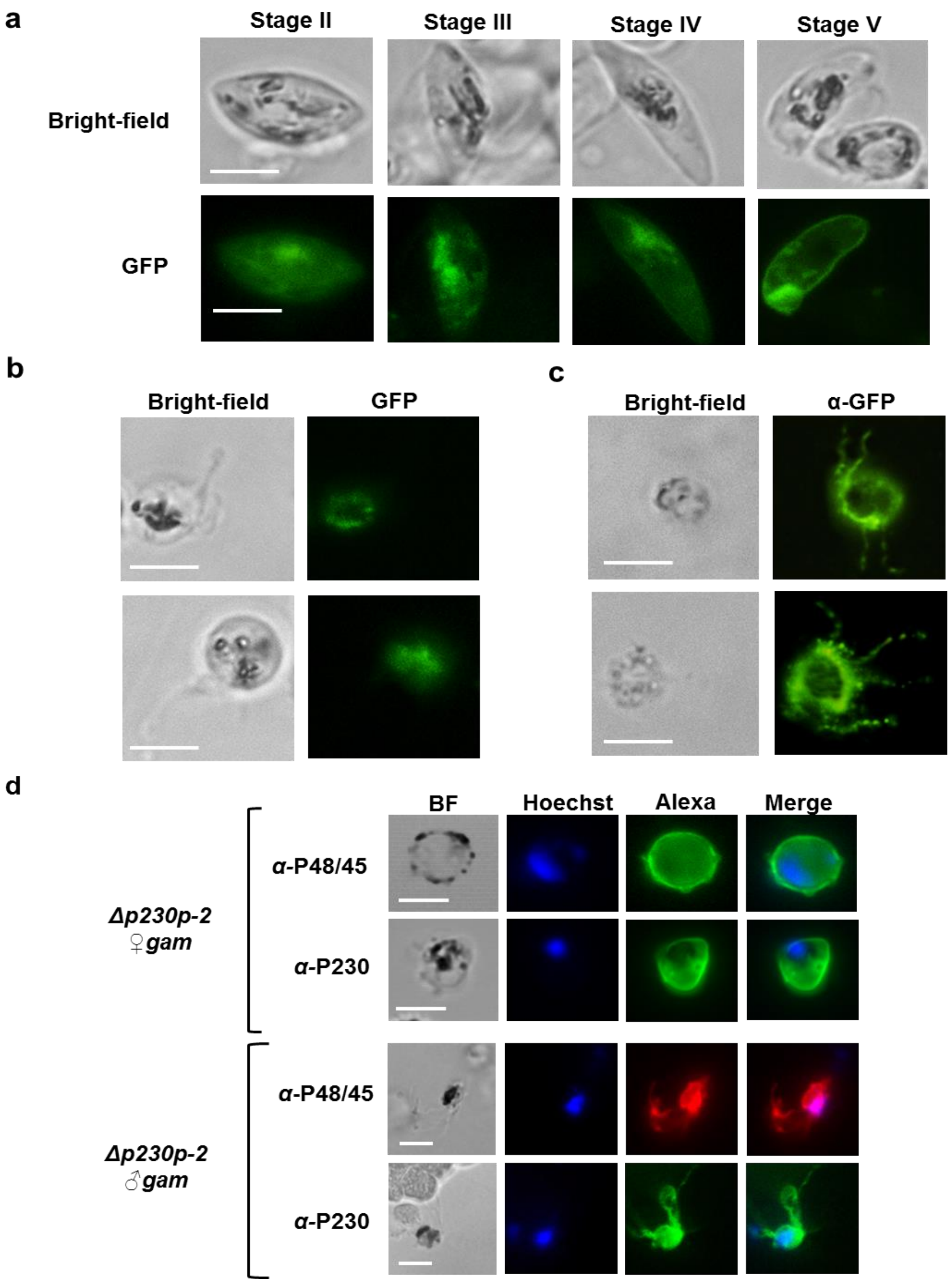

Suplementary Figure S1: Analysis of expression of P230p , P230 and P48/45 in mature and activated gametocytes of P230p-GFP parasites and *PfΔp230p-2*. a. GFP-fluorescence in different stages of gametocyte development, stage II, III, IV and mature, stage V, gametocytes of a transgenic *P. falciparum* (3D7) line that expresses a C-terminal GFP-tagged version of *p230p* (*p230p* -gfp). Scale bar, 7µm**. b.** GFP-fluorescence in live male gametocytes of the P230p-GFP line 15-20min after activation. Scale bar, 7µm. **c**. Immunofluorescence analysis of fixed activated male gametocytes of the P230p-GFP line 15-20min after activation. Cells were fixed with methanol and labelled with rabbit anti-GFP antibody followed by goat anti-rabbit antibody secondary conjugated to Alexa Fluor® 488. Scale bar, 7µm. **d.** Immunofluorescence analyses of mature, stage V, *PfΔp230p-2* gametocytes after activation with fetal calf serum. Upper panel, female gametes 30 minutes after gametocyte activation. Unfixed parasites were labelled with mouse anti-P230 (MAb 63F2A2) or rat anti-P48/45 (MAb 85RF45.1) antibodies followed by secondary conjugated antibodies (i.e. anti-mouse or anti-rat

IgG Alexa Fluor® 488 (green)). Nuclei stained with the DNA-specific dye Hoechst-33342. All pictures were recorded with standardized exposure/gain times; Alexa Fluor® 488 (green) 0.7 s; Hoechst (blue) 0.136 s; bright field 0.62 s (1x gain). Scale bar, 7µm. Lower panel, Immunofluorescence analyses of male gametes 15 minutes after gametocyte activation in fetal calf serum. Cells were fixed with methanol and labelled with mouse anti-P230 (MAb 63F2A2) or rat anti-P48/45 (MAb 85RF45.1) antibodies followed by secondary conjugated antibodies (i.e. anti-mouse IgG Alexa Fluor® 488 (green) or anti-rat IgG Alexa Fluor® 594 (red)). Nuclei stained with the DNA-specific dye Hoechst-33342. All pictures were recorded with standardized exposure/gain times; Alexa Fluor ® 488 (green) 0.7 s; anti-IgG Alexa Fluor ® 594 (red). 0.6s; Hoechst (blue) 0.136 s; bright field 0.62 s (1x gain). Scale bar, 7µm.

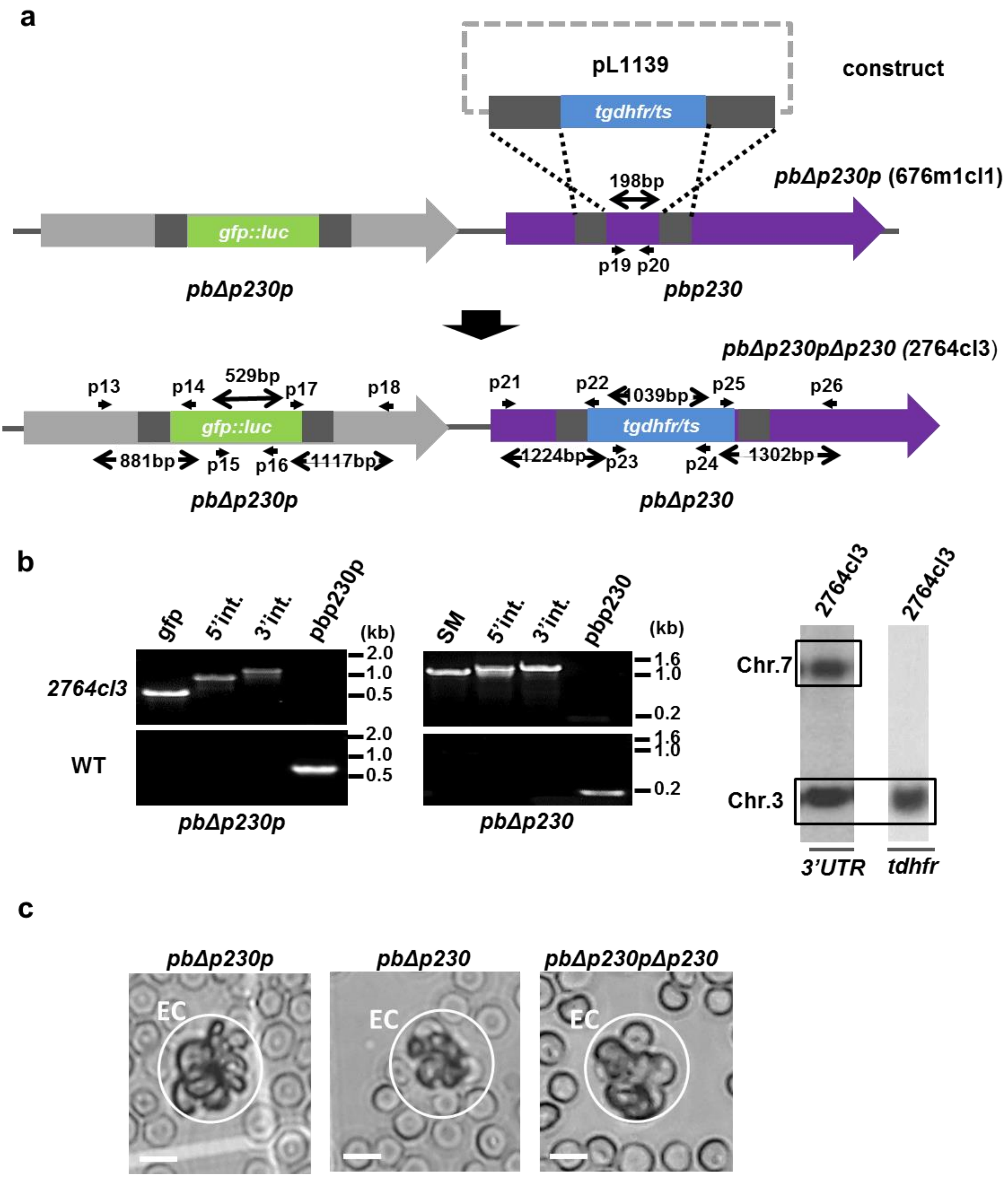

Suplementary Figure S2: Generation and analysis of a *P. berghei* mutant line (*pbΔp230pΔp230*) lacking expression of P230 and P230p. a. Schematic representation of the generation of the *P. berghei* double gene-deletion mutant *pbΔp230pΔp230*. To generate this mutant the *p230* locus was disrupted in the existing *PbΔp230p* mutant (676m1cl1) which has a disrupted *p230p* locus containing a GFP-Luciferase expression cassette. To disrupt *p230* a DNA construct (pL1139) was used, which integrates into the P. berghei genome by double cross-over integration and replaces (part of) the *p230* locus with the selectable marker (SM) cassette containing *Toxoplasma gondii* dihydrofolate reductase/thimidylate synthase (*tgdhfr/ts*). Parasites of line 676cl1 were transfected with this construct (exp. 2764) using standard transfection technologies and selection

with pyrimethamine. Selected parasites were cloned by limiting dilution and mutant 2764cl3 was used for genotype and phenotype analysis (see **b,c**). Location of primers (p) and PCR amplicons (in black) are indicated. Primer sequences are shown in **Supplementary Table S1**. **b.** Diagnostic PCR (left, middle panel) and Southern analysis of PFG-separated chromosomes (right panel) confirms correct disruption of *p230* and *p230p* in line 2764cl3. 5'and 3'integration PCR (int), shows the expected bands of 881 bp and 1117 bp for disruption of *p230p* (primers p13/p14 and p17/p18) and 1224 bp and 1302 bp for *p230* (primers p21/p22 and P25/26). The GFP-Luciferase cassette in *p230p* was detected with primers p15/p16 (529bp) and the *tgdhfr/ts* SM cassette in the *p230* locus with primers p23/p24 (1039bp). Uncropped images of the gel images are shown in **Supplementary Fig. S6**. See **A** for primers, PCR amplicons and **Supplementary Table S1** for primer sequences. Hybridization of PFG-separated chromosomes with the *tgdhfr* probe shows the integration of the *tgdhfr/ts* SM cassette in *230p* on chromosome 3. Hybridization with the probe against 3'UTR of the *P. berghei dhfr/ts* gene recognizes the endogenous *dhfr/ts* gene on chromosome 7 and the integrated casettes in *p230* and *p230p* in chromosome 3. Uncropped images of PFG-Southern analyses are shown in **Supplementary Fig. S**7. **c**. Exflagellation centres (EC, circles) as observed by light microscopy analysis of male gametocytes of *pbΔp230p* (line 676m1cl1), *pbΔp230* (line 310cl1) and pbΔp230pΔp230 (line 2764cl3) between 10 and 20 min after activation in live preparations in a Bürker cell chamber. Scale bar, 7µm. See also **Supplementary videos S7-S12** for the presence of exflagellation centres and **Supplementary Table S4** for quantification of exflagellation centres in the mutant and wild type parasites.

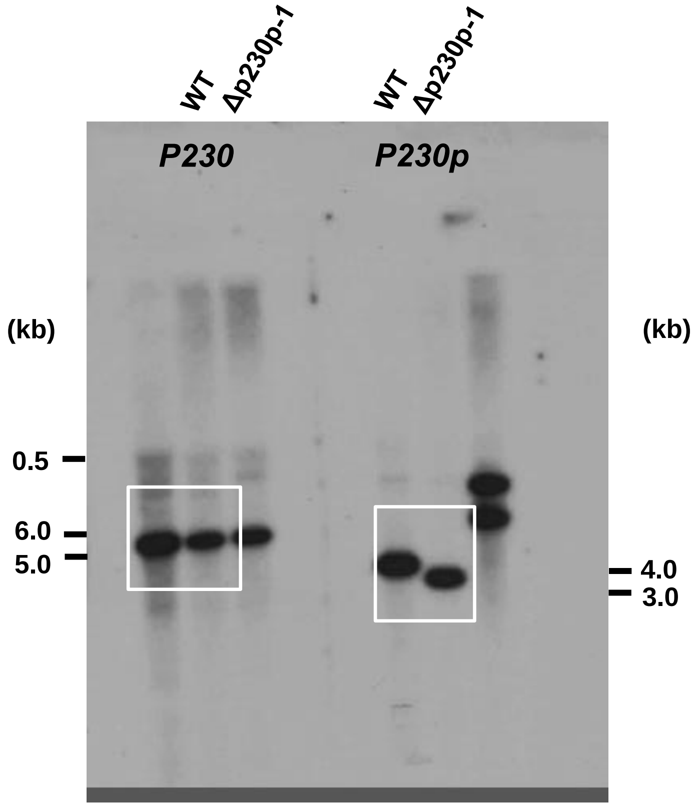

Supplementary Figure S3. Unprocessed images of Southern blot analysis. The white boxes show the cropped image in Fig.1b. Molecular marker 1Kb plus ladder (M). DNA was electrophoresed in 0,8% agarose gel.

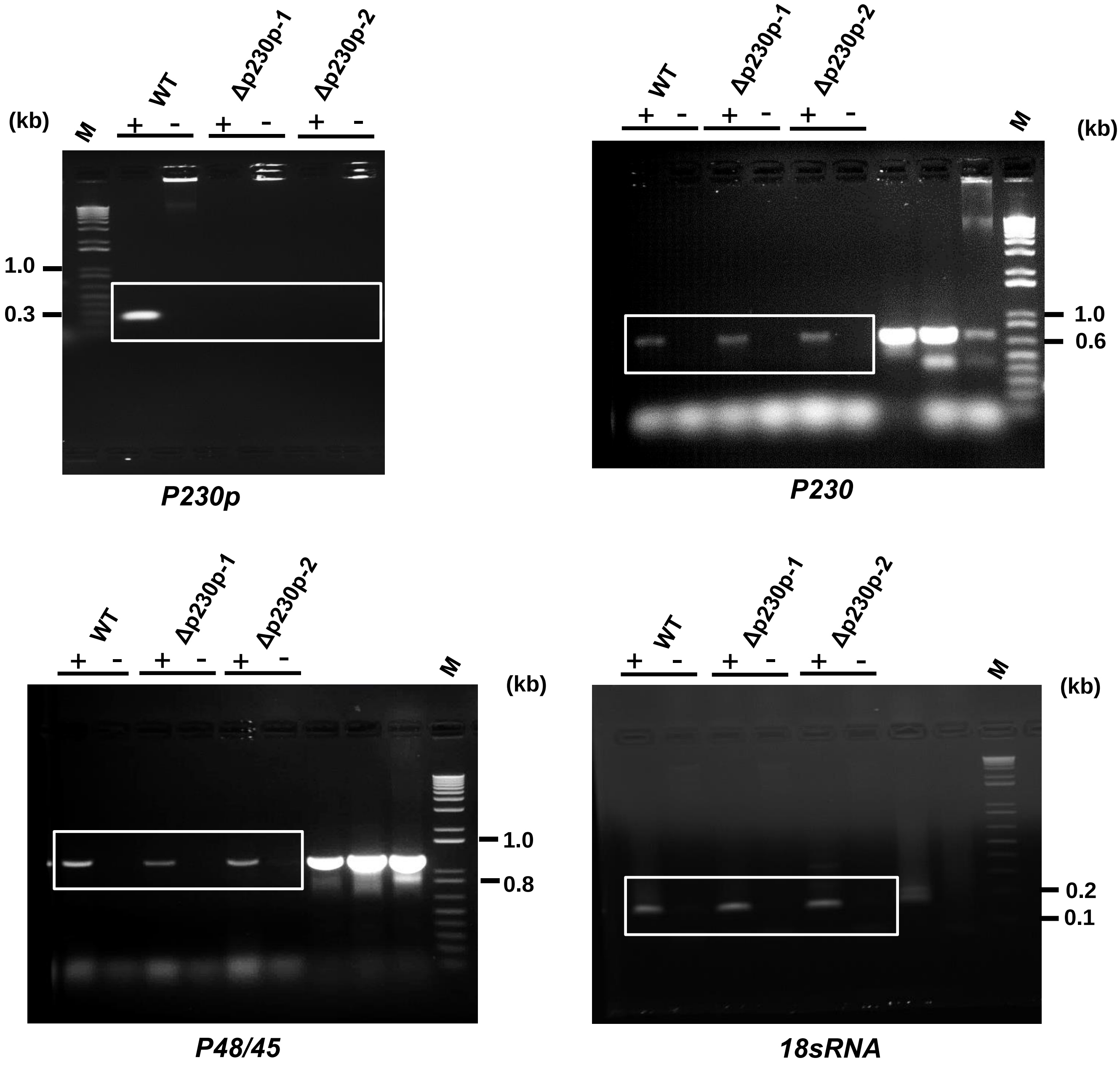

Supplementary Figure S4. Unprocessed images of RT-PCR analysis. The white boxes show the cropped image in Fig. 1c (left panel). Molecular marker 1Kb plus ladder (M). DNA was electrophoresed in 1% agarose gel.

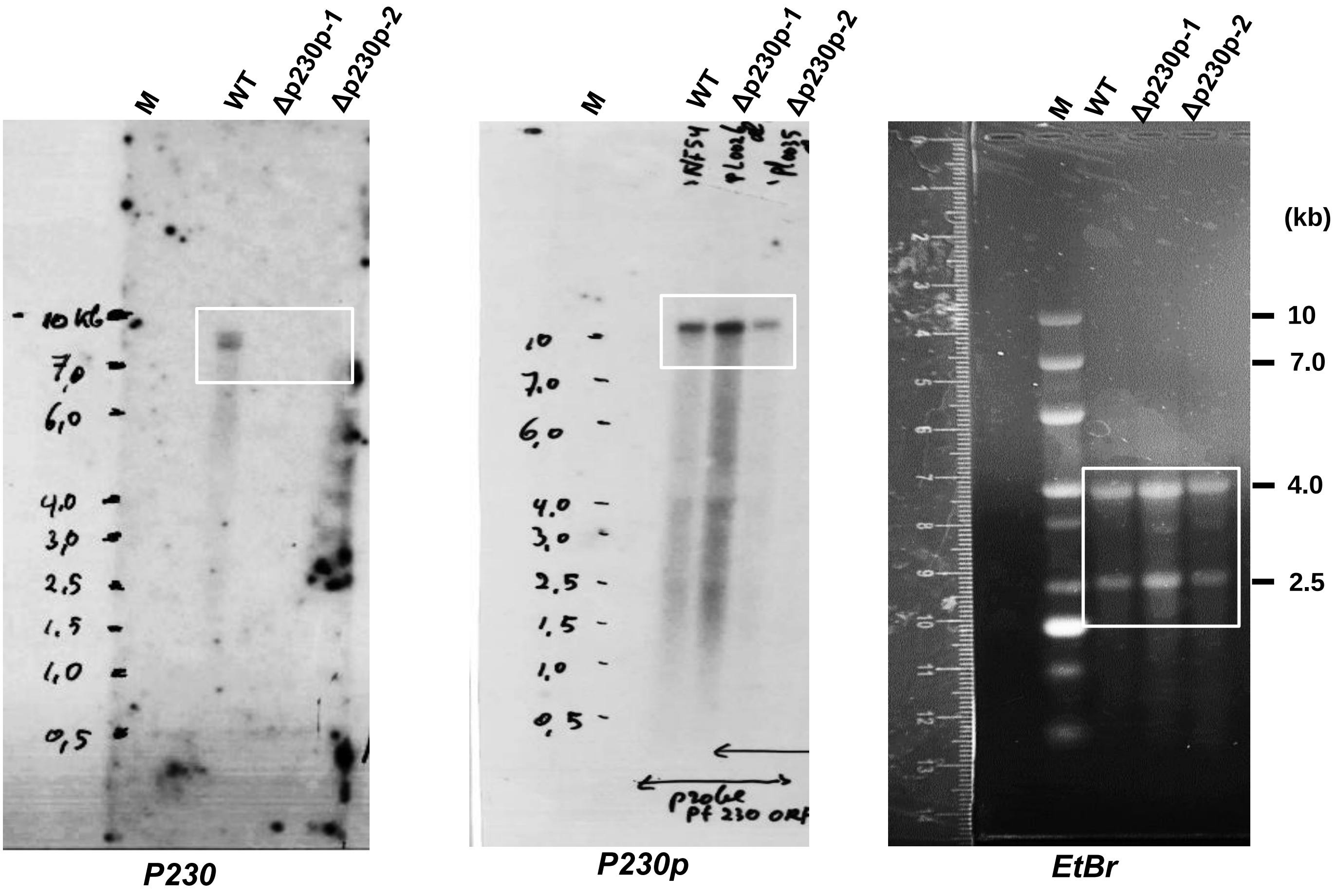

Supplementary Figure S5. Unprocessed images of Northern blot analyses. The white boxes show the cropped image in Fig.1c (right panel). P230 and P230p panels show autoradiograph images after probing with 230 or 230p probes. EtBr panel shows electrophoresis gel stained with ethidium bromide *(EtBr)* of WT and *PfΔ230p* parasites. 5ug of RNA was seeded per lane. RNA Molecular weight marker (M) and RNA was electrophoresed in 1% agarose gel.

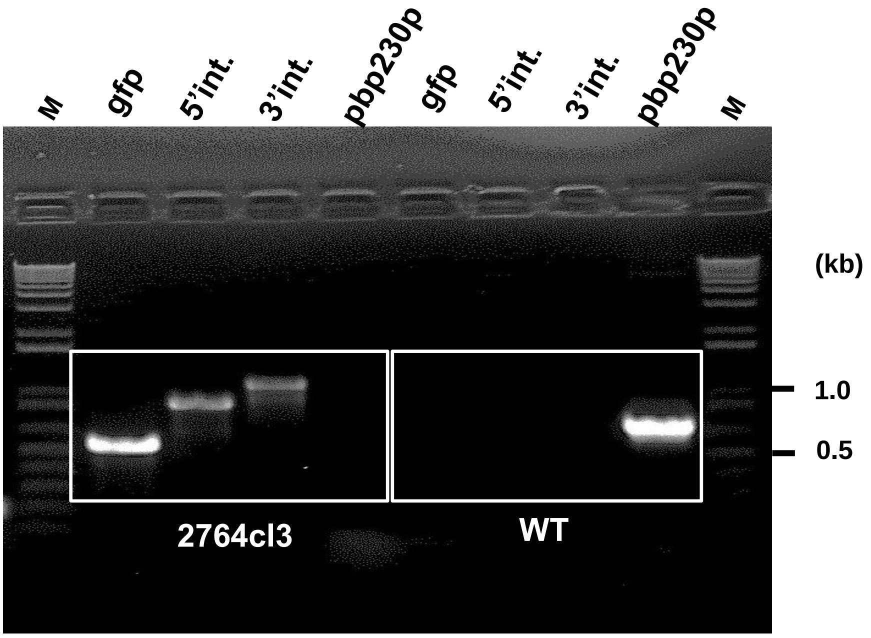

pbΔp230p

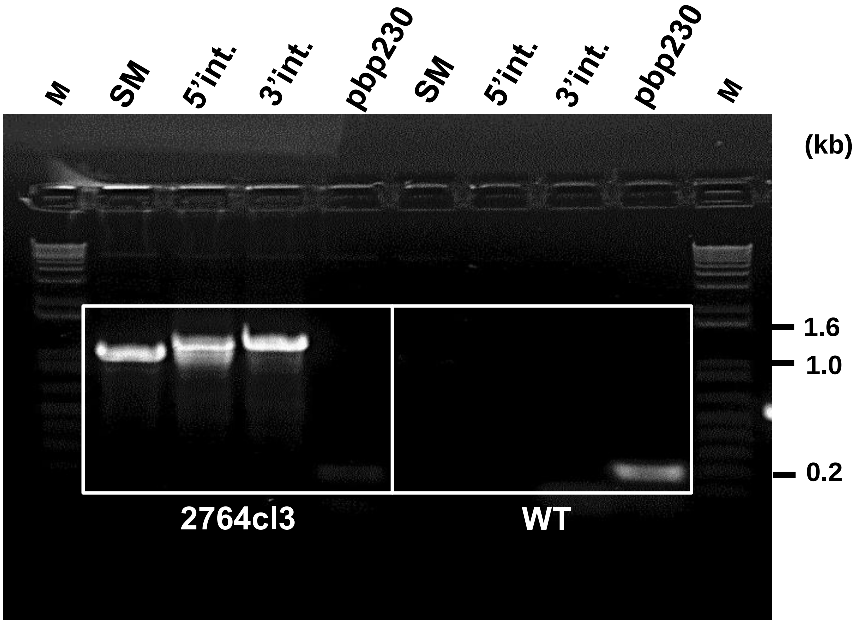

pbΔp230

Supplementary Figure S6. Unprocessed images of PCR analyses. The white boxes show the cropped image in Supplementary Fig.2 (Left and Middle panel). Molecular marker; 1Kb plus ladder (M). DNA was electrophoresed in 1% agarose gel.

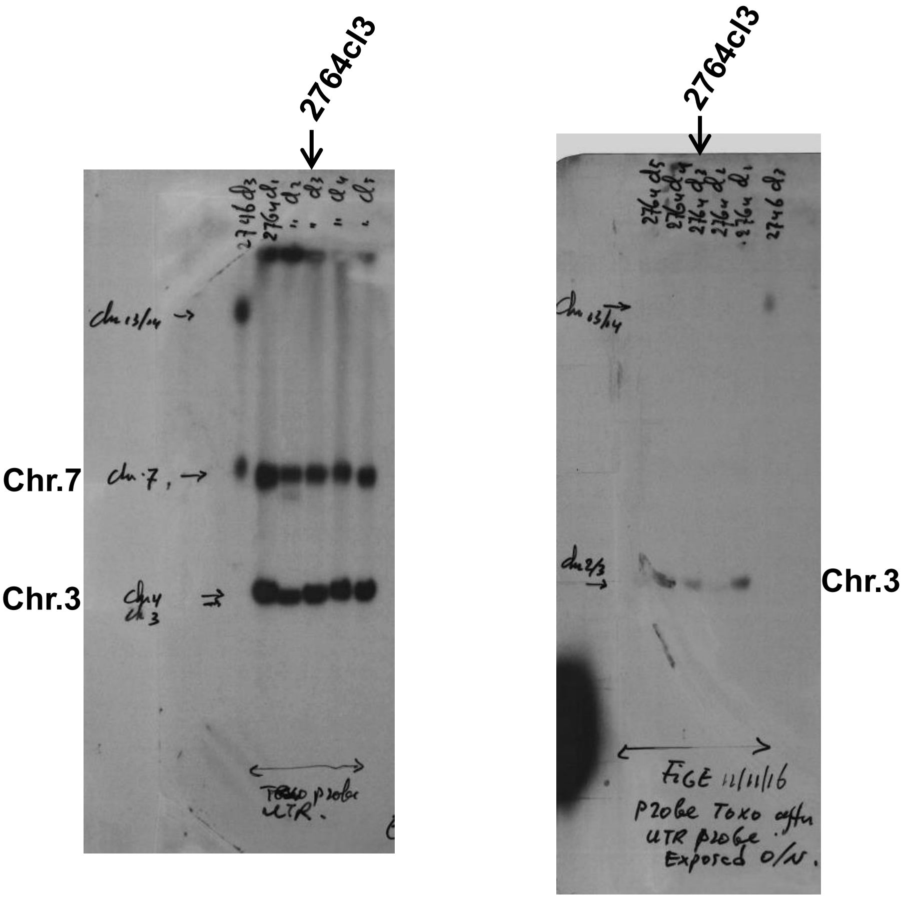

Supplementary Figure S7. Unprocessed images of (PFG-separated) chromosomal Southern Blot analyses. The arrows indicated the lanes cropped out of this image and used in Supplementary Fig. 2b (Right panel). DNA was electrophoresed in 1% agarose gel.

Supplementary Table S1. List of primers used in this study

Primer ID	Leiden code	Gene ID	Sequence	Product (bp)	Description
			pfΔp230p genotyping		
P1	8386	PF3D7_0209000	CGCTGAAGAATCTATTCCTC	745	Forward *pfs230*
P2	8387	PF3D7_0209000	CTATCCGAGGGGGTTAAATAC		Reverse *pfs230*
P3	7869	PF3D7_0208900	TTATTGGGCCCGTCGACGTTGATAAGGATAGTGTTTCAG	867	Forward HR2 *pf230p*
P4	7871	PF3D7_0208900	TCCTTAAGCTTTACGTAGGATTAATATTCCCATTAGG		Reverse HR2 *pf230p*
P5	8038	PF3D7_0208900	GATGATCTAAAAAAAGAAGAGTG	259	Forward *pf230p*
P6	8039	PF3D7_0208900	CATTACAATAACAAAATAAATGAAC		Reverse *pf230p*
P7	6068	PF3D7_1346700	ATTCCATATGAAAACAATGATTTTTGTAAGCCTAGC	1219	Forward *pfs48/45*
P8	6069	PF3D7_1346700	GGCGGCCGCTTACTATAGTAACTGTCATATAAGCAC		Reverse *pfs48/45*
P9	8733		GTTAAGGGAGTGAAGACGATCAGA	165	Forward *plasmodium 18sRNA*
P10	8734		AACCCAAAGACTTTGATTTCTCATAA		Reverse *plasmodium 18sRNA*
			pbΔp230pΔp230 Genotyping		
P11	8658	PBANKA_0306000	AATGCACCATCGTATGTGATAG	715	Forward *pb230p*
P12	8659	PBANKA_0306000	CGTCCCATCTATGCTACTCAC		Reverse *pb230p*
P13	5510		GCAAAGTGAAGTTCAAATATGTG	881	Forward 5' Integration *pbΔ230p*
P14	7289		TAAAGCACAATATCTAGGATACTAC		Reverse 5' Integration *pbΔ230p*
P15	7295		ATAAGAATGCGGCCGC GATCTATGAGTAAAGGAGAAGAAC	529	Forward gfp::luc reporter cassette
P16	7294		CTTCCATCTTCAATGTTGTGTC		Reverse gfp::luc reporter cassette
P17	7922		GTCTCTTCAATGATTCATAAATAGTTGG	1117	Forward 3'Integration *pbΔ230p*
P18	5511		AGTGACTTTCAGTGAAATCGC		Reverse 3'Integration *pbΔ230p*
P19	8654	PBANKA_0306100	CAACATGTACTTAAGTTAGACTTAG	198	Forward *pbs230*
P20	8655	PBANKA_0306100	GGATTCATTAAAATATTTTCCATATTTATG		Reverse *pbs230*
P21	8656		GCCCAACAAAAGATTTAGGAAAT	1224	Forward 5' Integration *pbΔs230*
P22	6382		ATTTGTGTCTATATTACCAACTC		Reverse 5' Integration *pbΔs230*
P23	4598		GGACAGATTGAACATCGTCG	1039	Forward tgdhfr/ts selectable marker
P24	4599		GTGTAGTCTGTGTGCATGTC		Reverse tgdhfr/ts selectable marker
P17	7922		GTCTCTTCAATGATTCATAAATAGTTGG	1302	Forward 3' integration *pbΔ230*
P26	8657		GCTTTCATATGTTGTATTAGTATTATCAC		Reverse 3' integration *pbΔ230*
P27	L692	PBANKA_0719300	CTTATATATTTATACCAATTG	561	Forward 3'UTR *pbdhfr*
P28	L693	PBANKA_0719300	GTTTTTTTTTAATTTTTCAAC		Reverse 3'UTR *pbdhfr*

Supplementary Table S2. Published RNAseq and proteome data on expression of P230, P230p and P48/45 in male (m) and female (f) gametocytes

[1] The ratio of RNAseq RPKM values in separated male and female gametocytes (Lasonder, E. et.al, 2016,

	Lasonder, E. et.al. 2016		Miao, J. et.al. 2017	Tao, D. et.al. 2014	Khan, SM.et.al 2005
**Protein**	**m/f ratio transcriptome[1]**	**m/f ratio proteome[2]**	**m/f ratio proteome[3]**	**m/f presence proteome[4]**	**m/f ratio proteome[5]**
P230p	54	54	male only	male only	male only
P230	4,4	0,5	1,1	in male and female	male only
P48/45	4,2	1,0	1,4	in male and female	4,1

Nucleic Acids Res 44(13):p.6087-101).

[2] The ratio of proteome spectra values in separated male and female gametocytes (Lasonder, E. et.al, 2016, Nucleic Acids Res 44(13):p.6087-101).

[3] The ratio of proteome spectra values in separated male and female gametocytes (Miao, J. et.al, 2017, Mol Cell Proteomics, 2017. **16**(4): p. 537-551.).

[4] The ratio of proteome spectra values in separated male and female gametocytes (Tao, D. et.al, 2014, Mol Cell Proteomics, 2014. **13**(10): p. 2705-24.).

[5] The ratio of proteome spectra values in separated male and female gametocytes (Khan, SM. et.al, 2005, Cell, 2005. **121**(5): p. 675-87).

Supplementary Table S3. Oocyst and sporozoite production in *A. stephensi* mosquitoes of WT and *pfΔp230p* parasites

Lines	No. of oocyst mean (range) [1]	No. of spz ($x10^3$) mean (range)[2]
WT		
NF54 (7 exp.)	18,73 (4-32)	56 (11-90)
Δp230p-1		
0022cl1 (6 exp.)	0,39 (0,05-0,6)	0
0022cl5 (5 exp.)	0,38 (0,1-0,7)	0
Δp230p-2		
0035cl4 (6 exp.)	0,12 (0-0,5)	0

[1] *Mean number of oocysts per mosquito at day 8 after feeding. Range corresponds to the mean number of oocyst in multiple experiments (5-7 exp. per line; 10-20 mosquitoes per exp.)*

[2] *Mean number of salivary gland sporozoites per mosquito at day 14 after feeding.*
Range corresponds to the mean number of oocyst in multiple experiments (5-7 exp. per line; 10-20 mosquitoes per exp.)

Supplementary Table S4. Exflagellation and fertilization rates of WT, *pbΔp230p*, *pbΔp230* and *pbΔp230pΔp230* parasites *in vitro*

Lines	% exflagellating males mean (SD)[1]	Exflagellation centers[2]	Fertilization rate (%) mean (SD)[3]
**WT**			
cl15cy1*	76-92 (85)	+++	59 (6,7)
pbΔp230p			
676m1cl1	50-92 (75)	+++	55-80(10)
pbΔp230			
310cl1*	72-90 (80)	+++	<0,1
323cl1*		+++	<0,1
pbΔp230pΔp230			
2764cl3 (n=2)	75-95 (85)	+++	<0,1 (0,01)

[1] Percentage of exflagellating male gametocytes between 10-20 minutes after activation.
[2] Presence of exflagellation centers between 10-20 minutes after activation. Exflagellation centers are counted in Bürker cel counter. +++: >90% of the exflagellating male gametocytes form exflagellation centers
[3] The fertilisation rate is the percentage of female gametes that develop within 24 hours into ookinetes in vitro.
* Data taken from van Dijk MR et.al, 2010, PLoS Pathog, 2010. **6**(4): p. e1000853
